# Interlinked Pathways: Exploring the Bidirectional Impacts of Periodontitis and Metabolic Syndrome

**DOI:** 10.7759/cureus.67544

**Published:** 2024-08-22

**Authors:** Bandar M Barnawi, Maram M Alanazi, Fai A Al-Mutiri, Rahaf S Alqahtani, Madhawi S Al-harbi, Saud K Al-Raqqas, Waleed K Mahjoub, Mahdi M Alsetri, Ziyad M Al-Sultan, Ghadeer M Alghamdi, Ridha I Almutawah

**Affiliations:** 1 Family Dentistry, Ministry of Health, Medinah, SAU; 2 College of Dentistry, King Saud Bin Abdulaziz University for Health Sciences, Riyadh, SAU; 3 Dentistry, King Abdulaziz Medical City Riyadh, Riyadh, SAU; 4 College of Dentistry, King Saud University, Riyadh, SAU; 5 Dentistry, Ministry of Health, Dammam, SAU; 6 Dentistry, King Faisal University, Alhfuf, SAU

**Keywords:** cytokines, insulin resistance, inflammation, metabolic syndrome, periodontitis

## Abstract

Metabolic syndrome (MBS) and periodontitis are distinct conditions with overlapping and unique risk factors. Periodontitis is a chronic destructive disease of the periodontium, driven by alterations in the host immune-inflammatory response to virulent periodontal pathogens. MBS is characterized by various abnormalities, including visceral abdominal obesity, dyslipidemia (low high-density lipoprotein (HDL) and high triglyceride (TG) levels), hypertension, and hyperglycemia. These factors collectively increase the risk of atherosclerotic cardiovascular disease (CVD) and diabetes. Several pro-inflammatory mediators are involved in the pathogenesis of periodontitis and MBS, and the deleterious bidirectional effects of these mediators exacerbate the severity and progression of both conditions. This comprehensive review focuses on the intricate relationship between MBS and periodontitis. Specifically, it explores the pathophysiological mechanisms of each disease component of MBS and its impact on periodontitis, and vice versa.

## Introduction and background

Periodontal disease (PD) is a low-grade chronic inflammatory disease, featuring a dysbiotic microbial community that causes a shift in the subgingival microbiota from Gram-positive to Gram-negative. This shift triggers the host immune-inflammatory system, leading to the destruction of the investing periodontal apparatus of the tooth [1-3]. The resultant persistent low-intensity inflammation by PD without intervention contributes to a characteristic systemic inflammatory state that has been linked to various systemic diseases such as cardiovascular diseases [4], obesity [5], insulin resistance (IR) [6], and metabolic syndrome (MBS) [7]. The inflammatory mediators that are released as a consequence of host-microbial interaction in PD include the proinflammatory cytokines such as interleukin (IL-1β) and tumor necrosis factor-alpha (TNF-α) that are upregulated in correlation with raised concentrations of prostaglandin E2 (PGE2) and elevated levels of C-reactive protein (CRP). The presence of these mediators is evident not only in periodontitis but also in systemic conditions like diabetes, obesity, and arthritis [8]. Around 20 to 60% of the global population is estimated to be impacted by varying degrees of periodontal disease [9-12], with approximately 7.4% of individuals, equivalent to 538 million people, suffering from a more severe form of periodontitis [13].

The sustained low-grade inflammatory condition may precipitate the emergence of IR and perturb the delicate balance of cytokine interactions within the periodontal tissues [14,15]. The induction of a proinflammatory state as a consequence of IR is purported to be a potential contributor to the development of MBS [16]. In 1988, Dr. Gerald Reaven introduced the term "Metabolic Syndrome", also known as "Reaven’s Syndrome" [17], "Insulin Resistance Syndrome" and "Syndrome X". This syndrome encompasses a collection of metabolic or systemic abnormalities, including hypertension, dyslipidemia, IR, and central obesity. The inflammatory state caused by these interconnected systemic abnormalities of MBS is associated with endothelial dysfunction which collectively elevates the likelihood of developing cardiovascular disease (CVD) and type 2 diabetes mellitus (T2DM) [18,19]. The potential linkage between metabolic syndrome and periodontal disease is posited to arise from their shared characteristic of being influenced by inflammatory responses, inflammatory cytokines, and heightened systemic oxidative stress [20]. The prevalence of metabolic syndrome demonstrates an age-dependent increase and exhibits variability across diverse ethnicities and genders within populations [21]. In contemporary times, metabolic syndrome has gained acknowledgment as a notable global epidemiological concern, with prevalence rates ranging from 17% to 32% in the general population [22].

This comprehensive review seeks to explore the correlation between MBS and periodontitis, focusing on elucidating the bidirectional impacts of the MBS cluster and periodontitis, including factors such as inflammatory responses, oxidative stress, biochemical changes, and microbiological effects. Additionally, it will investigate common risk factors shared between MBS and periodontitis, alongside individual discussions on the components of MBS, incorporating the latest evidence from the literature.

## Review

Research methodology

A comprehensive search was conducted on digitized databases, including PubMed, Embase, Medline, and Google Scholar, utilizing specific keywords such as “periodontitis,” “metabolic syndrome,” “insulin resistance,” “hyperglycemia,” “obesity,” “dyslipidemia,” and “hypertension.” The initial screening involved identifying relevant articles through title and abstract reviews, followed by full-text evaluations of the selected studies.

The inclusion criteria for this review comprised narrative reviews, systematic reviews, randomized controlled trials, comparative studies, clinical trials, and case series focusing on the bidirectional impacts of periodontitis and metabolic syndrome. Studies that examined inflammatory markers, microbial changes, systemic impacts, and patient outcomes related to both conditions were included.

Conversely, studies that focused solely on surgical interventions, antimicrobial therapies, or conditions unrelated to periodontitis and metabolic syndrome were excluded. Additionally, articles that did not provide specific data on the interaction between periodontitis and metabolic syndrome or those lacking sufficient methodological detail were omitted from this review.

Overview of periodontitis as an inflammatory disease

Severe periodontitis is recognized as the sixth most commonly occurring disease worldwide and represents a significant contributor to disability-adjusted life years within oral health conditions [23]. The etiology of periodontal disease involves a multifactorial interplay of bacterial plaque and dental biofilm, microbial by-products, the host's immune response, environmental and behavioral influences, and genetic predispositions [24]. While the presence of bacteria is a fundamental element in the progression of periodontitis, it is not solely responsible for the onset of the disease. Subgingival microorganisms and their deleterious products, such as lipopolysaccharides (LPS), reach the systemic circulation and the investing periodontal tissues through ulcerations of the sulcular epithelium, stimulating macrophages to secrete cytokines such as IL-1α, IL-1β, and TNF-α [1,25].

In the absence of disease-modifying risk factors, the body typically responds in a way that limits bacterial buildup and mitigates the likelihood of infection. Coexisting comorbidities such as diabetes and smoking can disrupt this normal immune-inflammatory response and drive it beyond its usual physiological limits. In the context of immune responses, both the antibody-mediated and cell-mediated pathways are activated, but the adverse effects are caused by disrupted repair processes or exaggerated inflammatory reactions [26]. Elevated levels of cellular and cytokine-mediated inflammatory markers, including CRP [27], fibrinogen [28,29], matrix metalloproteinases (MMPs) [30], and various cytokines [31], have been linked to periodontitis.

In PD, the bacterial load interacts with the innate immune system through mechanisms that involve the recruitment of neutrophils, macrophages, monocytes, and mast cells to the site of infection. This process triggers elevated levels of cytokines in response to bacterial invasion, contributing to recurrent cell recruitment and complement activation, which in turn leads to additional tissue damage. PGE2, a hormone with local effects, induces the release of MMPs from macrophages, fibroblasts, and endothelial cells, resulting in the degradation of the extracellular matrix. This leads to a cascade of events [24], where TNF-α, PGE2, IL-1β, IL-6, IL-11, and IL-17 elevate the activity of receptor activator of nuclear factor kappa-B ligand (RANK-L). The interaction of RANK-L with its receptor RANK activates a pathway that triggers osteoclast differentiation, ultimately resulting in crestal bone loss [1,24,32].

The inflammatory processes and immune responses involved in periodontitis have broader implications for systemic health, particularly in the context of metabolic conditions. One such condition is metabolic syndrome, which shares several overlapping risk factors and pathogenic mechanisms with periodontitis.

Metabolic syndrome

MBS is a collective cluster of conditions that increase the risk of developing CVD and T2DM. Obesity, insufficient physical activity, and IR are the primary risk factors for MBS. Factors such as advancing age, genetic predisposition, and hormonal imbalances further increase this risk [14].

The diagnostic criteria for MBS are primarily based on several core components: central obesity, low high-density lipoprotein (HDL) cholesterol, elevated triglyceride (TG) levels, increased blood pressure, and elevated blood glucose levels [20]. Various professional organizations have developed distinct sets of diagnostic criteria for MBS, but all of them emphasize these fundamental components. Initially, the widely adopted framework was the National Cholesterol Education Program's Adult Treatment Panel III (NCEP ATP III) [20,33], later revamped by the American Heart Association (AHA) and the National Heart, Lung, and Blood Institute (NHLBI) in 2005 [14]. Both iterations require the presence of at least three out of the five aforementioned components for a metabolic syndrome diagnosis.

Additionally, the International Diabetes Federation (IDF) and the World Health Organization (WHO) formulated specific diagnostic guidelines of their own. The IDF criteria notably highlight central obesity as a pivotal criterion, distinguishing it from the criteria used by the NCEP ATP III and AHA/NHLBI [14]. Conversely, the WHO criteria states glucose intolerance, impaired glucose tolerance, or diabetes, alongside IR, as integral components for diagnosing MBS [34]. For standardization purposes, metabolic syndrome is defined as the presence of three out of the aforementioned five interconnected conditions [18]. The predominant components characterizing metabolic syndrome commonly include abdominal adiposity, hypertension, and hyperglycemia [35].

Correlation between metabolic syndrome and periodontal disease

The primary connection between MBS and periodontal disease lies in the interaction between oxidative stress and microbial dysbiosis, resulting from their similar inflammatory characteristics [20,36]. In periodontal disease, pro-inflammatory cytokines migrate from the gingiva into the vascular circulation, thus elevating oxidative stress. This worsens IR and promotes atherosclerotic changes, which predispose individuals to metabolic syndrome. Similarly, in metabolic syndrome, inflammatory cytokines may intensify gingival oxidative stress and, therefore, weaken the periodontium's capacity to handle bacterial assaults. This increases susceptibility to periodontal disease, producing a reciprocal association [20].

Obesity and type 2 diabetes can alter the composition of the oral microbiome, resulting in a state of microbial imbalance [36]. Obese individuals with periodontal disease exhibit distinct differences in their oral microbiome composition compared to their non-obese counterparts. Specifically, obese patients demonstrate reduced oral microbial diversity, and this could increase their vulnerability to periodontal disease [37].

Two hypotheses exist regarding the correlation between periodontitis and metabolic syndrome: one suggests a causal relationship, while the other emphasizes common risk factors. Both conditions share risk factors like hyperglycemia, obesity, dyslipidemia, and high blood pressure, along with confounding elements such as excessive calorie consumption, lack of physical activity, and poor oral hygiene. Evidence also indicates a reciprocal connection, where inflammatory markers in metabolic syndrome can worsen periodontal inflammation and vice versa [1,38,39].

In a previous study, subjects diagnosed with MBS displayed elevated concentrations of plasminogen activator inhibitor type-1 (PAI-1) compared to their healthy counterparts [40]. The involvement of PAI-1, along with tissue plasminogen activator, is integral to the pathogenesis of periodontitis through the modulation of extracellular matrix proteolysis. Furthermore, obesity can impact PAI-1 levels, potentially exacerbating the progression of periodontitis [1,41]. Accordingly, Nishimura et al. proposed that periodontal disease should be considered a component of MBS [42].

Gomes-Filho et al. [43] conducted a case-control study and found that individuals with moderate to severe periodontitis had a twofold increased risk of MBS compared to those without the condition. This increased risk was higher in severe cases, indicating a positive association between the severity of periodontitis and MBS. In their systematic review, Rosário-dos-Santos et al. [44] elucidated that moderate and severe levels of periodontitis are associated with MBS, indicating a potential dose-response relationship. In addition, Pham et al. [45] discovered that a substantial proportion (21%) of individuals diagnosed with metabolic syndrome had severe periodontitis, compared with the lower prevalence (6.8%) observed among healthy counterparts. Moreover, elevated levels of periodontal indicators, including bleeding on probing (BOP), gingival index (GI), plaque index (PI), probing pocket depth (PPD), and clinical attachment level (CAL), were noted among those with metabolic syndrome. They observed a correlation between the number of metabolic syndrome components and the worsening severity of periodontal disease, with those having four to five components displaying the poorest periodontal parameters. Understanding metabolic syndrome is crucial, as its components significantly influence and are influenced by various chronic conditions, including periodontitis. A key factor in this relationship is insulin resistance, a cornerstone of metabolic syndrome and a significant player in periodontal disease.

Insulin resistance and periodontitis

IR is a metabolic disorder characterized by a decrease in the effectiveness of insulin on tissues in response to normal levels of insulin. It results in elevated blood glucose levels and is classified as a precursor to diabetes. Prediabetes is recognized as a component of MBS due to its correlation with IR, and it is also a strong indicator of developing T2DM [33,46].

MBS is often attributed to the effects of IR, which initiates a pro-inflammatory state. IR arises when the pancreas produces insulin that is incompletely utilized by muscle, fat, and liver cells due to impaired signaling, consequently diminishing glucose metabolism from the bloodstream [47]. As a result, inflammation occurs due to various mechanisms, such as oxidative stress, increased concentration of free fatty acids, and interactions with the anti-inflammatory effects of insulin [48]. Advanced glycation end products (AGEs) interacting with their receptors (RAGEs) promote local oxidative damage and act as markers for systemic oxidative stress, which is heightened in MBS [49]. Furthermore, enlarged waist circumference and elevated body mass index (BMI) are closely associated with IR; these factors are linked to visceral adipose tissue accumulation and enhanced adiposity. Subsequently, adipocytes and macrophages in adipose tissue produce cytokines, such as TNF-α and IL-6. These molecules are associated with pro-inflammatory activity and are involved in the development of IR [50], with elevated circulating levels of TNF-α and IL-6 found in subjects with obesity and IR [51].

Researchers have found correlations between IR and periodontal disease. In one study, individuals previously diagnosed with IR showed a markedly higher occurrence of moderate to severe periodontal disease compared to their healthy counterparts [52]. One potential explanation for the link between periodontal disease and IR could be the inflammatory process [53]. IR is characterized as a low-intensity, chronic inflammatory state. In cases of IR, there is an increase in pro-inflammatory molecules like interleukin IL-1, IL-6, and TNF-α [54,55]. Another inflammatory agent that may be associated with the condition is reactive oxygen species (ROS) [56].

The relationship between IR and periodontal disease is believed to be reciprocal. Periodontal disease is characterized by chronic inflammation, leading to the release of pro-inflammatory cytokines in the gingiva, which can enter the bloodstream and potentially exacerbate preexisting IR or contribute to the onset of diabetes mellitus [46]. In addition to the inflammatory pathway, the microbial approach is also implicated in the association between IR and periodontitis. In an in vitro study, lipopolysaccharides from Porphyromonas gingivalis were observed to have a significant influence on the advancement of β-cell compensation and IR among individuals in the prediabetic stage with periodontitis [57]. Another critical aspect of metabolic syndrome that closely interacts with periodontitis is hyperglycemia, particularly in the context of type 2 diabetes mellitus.

Hyperglycemia and periodontitis

T2DM is a prevalent metabolic disorder characterized by impaired regulation of glucose levels in the body. This condition arises from a continual decline in the functioning of β cells and high levels of glucose in the blood. The inability of β cells to adequately counteract IR leads to the development of T2DM, making IR a key element of T2DM [58].

Hyperglycemia is the most well-documented element of metabolic syndrome in association with periodontal disease. The proposed explanations for this relationship focus on the release of AGEs, oxidative stress, and inflammation. Elevated levels of oxidative stress and ongoing subclinical inflammation throughout the body hinder the regulation of blood sugar levels and promote IR, thereby creating a pathway for the development of T2DM [59,60]. AGEs then build up due to the non-enzymatic glycation of proteins by persistently high blood sugar levels, leading to increased oxidative stress in the periodontium and higher levels of RANKL expression. AGEs also stimulate macrophages to produce inflammatory cytokines, which play a crucial role in triggering the release of acute-phase reactants such as C-reactive protein, thereby worsening the existing inflammation [61].

Individuals who have diabetes and severe periodontitis exhibit impaired chemotaxis, phagocytosis, and altered superoxide production by polymorphonuclear leukocytes (PMNs). Subsequently, killing capacity is compromised, and dysfunctional PMNs accumulate in periodontal tissues, ultimately causing an abscess-like state [56,62,63]. Individuals diagnosed with T2DM with periodontitis typically exhibit elevated biomarkers and oxidative stress markers. This raised oxidative stress may detrimentally impact β-cell function and further exacerbate IR. In periodontitis, elevated oxidative stress arises from an exaggerated neutrophil response that culminates in the increased release of ROS. Furthermore, there is evidence of diminished plasma antioxidant capacity in this population [6,64].

Metabolic pathways such as the polyol pathway [65], the hexosamine pathway [66], and the activation of protein kinase C (PKC) [67] elevate oxidative stress during hyperglycemia. Pro-inflammatory cytokines such as TNF-α, IL-1β, IL-6, and IL-18, elevated due to IR caused by diabetes, contribute to general body inflammation, thereby influencing bone loss and periodontal breakdown [46,66]. MMPs activated by these cytokines coalesce with ROS, leading to periodontal breakdown and collagen deterioration [68,69]. |Diabetes increases the ratio of RANKL/OPG and paves the way for osteoclastogenesis activation, resulting in deeper pockets, increased probing depth, and periodontal bone loss [69].

Patients with diabetes show reduced oral biological and phylogenetic diversity [70-72] as well as less species diversity. However, they have a higher abundance of periodontopathic microorganisms than healthy controls [70,72-74]. A similar pattern was observed in the subgingival microbiome environment, indicating that although patients with diabetes may appear to have a healthy periodontium, they have a higher risk of developing periodontal disease [72-74].

Quadri et al. conducted research in Saudi Arabia to examine the potential reciprocal correlation between periodontitis and T2DM. Their findings demonstrated a significant link between T2DM and hypertension among individuals with periodontitis. Numerous other studies have reported a moderate decline of 0.4% in the average hemoglobin A1C (HbA1C) level among diabetic individuals undergoing non-surgical interventions for periodontal disease [75,76]. After three months, diabetic subjects undergoing scaling experienced an average reduction in HbA1c levels of 0.3%. This is clinically significant, as a mere 1% drop in HbA1c levels has been linked to a remarkable 21% drop in diabetes-related mortality and a 37% drop in microvascular complications [75]. In 2023, a systematic review and meta-analysis evaluated the impact of poorly controlled diabetes mellitus on the development or progression of periodontitis; it showed an 86% increased risk associated with poorly managed diabetes mellitus, indicating an increased vulnerability to periodontitis among individuals with diabetes [77]. Alongside hyperglycemia, obesity is another critical factor within metabolic syndrome that exerts substantial effects on periodontal health.

Obesity and periodontitis

Obesity, a chronic multifactorial disease, is an imbalance of energy metabolism that manifests as an accumulation of excess fatty tissue posing a health risk [78-80]. The BMI is the primary method of measuring body fat [81]. A BMI exceeding 30 kg/m² is classified as obese, while a BMI between 25 and 29.99 kg/m² is categorized as overweight. An overweight individual is defined as having a waist circumference greater than 90 cm for women or 100 cm for men. An obese individual is characterized as having a waist circumference exceeding 105 cm for women or 110 cm for men [82]. In 2022, the WHO reported a concerning rise in obesity across Europe post-COVID-19, particularly among adolescents and children [83]. The buildup of fat tissue, especially around the abdomen (abdominal obesity), is linked to more than 200 health complications, such as T2DM, hypertension, and dyslipidemia, all of which are key components of MBS [84].

The connection between obesity and periodontal disease is increasingly evident in those with higher BMIs [85]. Increased waist circumference, weight gain, obesity, and being overweight are potential risk factors for periodontitis [5,86,87]. Adipose tissue is actively involved in influencing inflammation and immunity; it secretes both pro- and anti-inflammatory molecules, including adiponectin, cytokines, and chemokines, in a disrupted or modified manner [88]. The hallmark of inflammation induced by adipose tissue in obesity is its impact on multiple organs such as the pancreas, liver, skeletal muscle, heart, and brain [89]. The significant role of adipose tissue in systemic inflammation in obesity is linked to its secretion of hormones and cytokines, which play a crucial role in the development of various comorbidities associated with obesity [90].

As mentioned earlier, adipocytes and macrophages within adipose tissue secrete TNF-α and IL-6, which influence the relationship between obesity and periodontitis [91,92]. These cytokines, often termed adipocytokines, modulate systemic inflammation by influencing insulin sensitivity and glucose metabolism via chemokine signaling pathways. IL-6 contributes to reduced insulin signaling, increased fatty acid oxidation, and elevated hepatic synthesis of CRP. Conversely, TNF-α promotes lipolysis and upregulates IL-6 expression in adipocytes and processes associated with IR [93,94]. The development of IR and periodontitis may be significantly influenced immunologically by these adipokines. According to Han et al. [95], the best marker to evaluate the correlation between obesity and periodontitis is the visceral fat area, thus indicating obesity as a major risk factor for periodontitis.

Obesity increases the presence of ROS, which in turn triggers the activation of chronic inflammatory mediators within the gingiva, including the cytokines discussed earlier. This process leads to bone loss, deeper probing depths, and attachment loss in periodontal tissues [96]. Furthermore, obesity could disrupt the development of osteoblasts due to the common origin of adipocytes and osteoblasts from pluripotent bone marrow stem cells (BMSC) [97]. The levels of frizzled-related protein 1, an inhibitor of Wnt/β-catenin signaling, are elevated in mild obesity but decreased in morbid obesity. This leads to an increase in marrow adipose due to the inhibition of the Wnt/β-catenin pathway that drives BMSC differentiation towards osteoblasts [98].

Obesity also affects the composition of the oral and subgingival microbiome. One study revealed that individuals who are overweight or obese have higher levels of Tannerella forsythia in their subgingival biofilms compared to individuals of normal weight [99]. Additionally, individuals with obesity displayed increased levels of T. forsythia, Fusobacterium spp., and Porphyromonas gingivalis in their saliva, irrespective of their periodontal health status [100,101]. Dyslipidemia, characterized by abnormal lipid profiles, also plays a pivotal role in the relationship between metabolic syndrome and periodontitis.

Dyslipidemia and periodontitis

Dyslipidemia is characterized by elevated levels of TG and slightly increased levels of low-density lipoprotein (LDL) with decreased levels of HDL, resulting in an anomalous lipid profile state that predisposes individuals to CVD. Elevated TG levels and decreased HDL levels are elements of MBS, and these factors are interconnected with periodontitis, establishing a bidirectional link [20]. The simultaneous presence of dyslipidemia and periodontitis could potentially be linked to oxidative stress and prolonged systemic inflammation. However, additional research is necessary to elucidate the exact mechanism behind this association [102].

The correlation between periodontitis and dyslipidemia could potentially be influenced by circulating pro-inflammatory cytokines in the bloodstream. It is suggested that periodontitis is not solely associated with decreased lipid metabolism but also contributes to the worsening of hyperlipidemia by increasing the expression of pro-inflammatory cytokines in both serum and gingival crevicular fluid [103].

Changes in lipid and lipoprotein profiles caused by infection or inflammation may result from a variety of mechanisms. These encompass enhanced release of very low-density lipoprotein (VLDL), elevated breakdown of fats in adipose tissue (lipolysis), raised production of fatty acids in the liver, decreased oxidation of fatty acids, and prolonged removal of VLDL due to decreased function of lipoprotein lipase and apolipoprotein-E [104-106]. Nakarai et al. observed that the presence of LPS triggered a rise in the lipolytic function of adipocytes when coexisting with macrophages. Consequently, they hypothesized that periodontal infection could prompt lipolysis and consequently increase the levels of circulating TG [107]. The inhibition of TG clearance or enhancement of their synthesis by LPS and cytokines could potentially play a role in the development of hypertriglyceridemia [108]. Similarly, Arreguin-Cano et al. noted that individuals diagnosed with periodontal disease exhibited elevated blood TG levels, reduced HDL levels, and deteriorated metabolic parameters [109].

During the initial primary stage, the changes in lipids and lipoproteins that occur in response to infectious or inflammatory situations are advantageous to the host due to their involvement in the innate immune system response. For instance, lipoproteins can counteract the effects of bacterial LPS by facilitating its rapid elimination from the bloodstream, diverting it away from monocytes and macrophages, decreasing the activation of immune cells, and reducing the production of cytokines [110]. However, the process of increasing the removal of excess cholesterol from peripheral tissues and its transfer to the liver for elimination is supported by HDL. Apart from this function, HDL also poses properties such as antibacterial, antioxidant, and anti-inflammatory effects. Consequently, a decrease in HDL levels along with an increase in triglycerides and LDL levels leads to a heightened pro-inflammatory state. Additionally, hyperlipidemia induces changes in the body's response to inflammatory stimuli from periodontal pathogens [111].

Serum antibodies against Porphyromonas gingivalis, a widely recognized pathogen associated with periodontitis, are more common in individuals with reduced levels of HDL [112]. Additionally, serum antibodies targeting Aggregatibacter actinomycetemcomitans (A. actinomycetemcomitans) and P. gingivalis were demonstrated to be increased in individuals with higher levels of LDL cholesterol [113]. In a previous study, mice that were subjected to a high-fat diet and developed metabolic syndrome experienced an increase in the formation of osteoclasts (i.e., osteoclastogenesis), leading to a loss of bone in the alveolar region [114]. The increase in the production of certain pro-inflammatory cytokines such as RANKL, IL-6, macrophage colony-stimulating factor, and monocyte chemoattractant protein-1 was associated with the process of osteoclast formation and the exacerbation of bone loss [114].

The development of systemic inflammation, triggered by the presence of bacteremia and systemic LPS from periodontal disease, is believed to initiate the synthesis of cholesterol. Moreover, a decrease in the levels of pro-inflammatory cytokines in the bloodstream was observed when periodontal treatment was combined with antihyperlipidemic therapy [115]. Lei et al. [116] also noted that a host's susceptibility to periodontitis is increased by hyperlipidemia, which hinders an appropriate immunological response to bacterial damage. Lipids can also disrupt enzyme systems and cell membrane-bound receptors located within cell membranes, leading to the development of periodontal disease. The toll-like receptor (TLR)-2 ligand in isolation did not result in a discernible increase in the tartrate-resistant acid phosphatase-positive cell population. However, a notable escalation in osteoclastogenesis was observed upon the concurrent exposure of TLR-2 with oxidized LDL [117]. Another significant metabolic syndrome component, hypertension, also shares a bidirectional relationship with periodontitis.

Hypertension and periodontitis

Hypertension is a widespread chronic vascular disease that poses a substantial risk to cardiovascular health, characterized by systolic blood pressure surpassing 140 mmHg or diastolic blood pressure exceeding 90 mmHg [1,118]. Periodontitis has been linked to enhanced atherosclerosis [119-121]. The inflammatory response initiated by periodontitis at a systemic level has the potential to contribute to the development of atherosclerosis. This systemic inflammation can result in arterial stiffening and faster pulse wave velocity, both of which serve as indicators of compromised arterial health. The resulting arterial stiffness, caused by the deterioration of the elastic properties of larger arteries, may be involved in the initiation or progression of hypertension [122-125]. Damage can occur to endothelial cells due to periodontal pathogens and their endotoxins, leading to atherogenesis and thrombus formation. The presence of inflammatory mediators from the periodontium can further exacerbate this, elevating the risk of CVD and hypertension [126]. In the pathogenesis of CVD and atherosclerosis, the endothelial inflammatory state induced by toxins discharged from oral bacterial load infiltrating the bloodstream via periodontal pockets serves as a primary stage. Hypertension may emerge as a potential consequence in later stages [127]. A recently conducted research investigation demonstrated that individuals with hypertension exhibited elevated levels of P. intermedia, P. gingivalis, and F. nucleatum compared to patients with normal blood pressure [128]. Interventional research has indicated that periodontal therapy can improve initial indicators of atherosclerosis, including endothelial function, carotid artery intima-media thickness, and pulse-wave velocity [121,129]. According to studies, patients with severe periodontal disease had greater left ventricular mass [122,130-132]. Another study revealed that the application of periodontal therapy led to a notable reduction of 12.5 mmHg in systolic blood pressure and 10 mmHg in diastolic blood pressure. Additionally, six months post-treatment, there was a decrease in CRP and IL-6 levels, and left ventricular mass decreased by 12.9 g [133]. Hypertension is a key factor in the development of metabolic syndrome [72]. A specific study found a connection between the occurrence of hypertension and the intensity of periodontal disease [134]. Another study illustrated a favorable association between hypertension and periodontal disease. Moreover, it suggested an increased vulnerability to the development of periodontal disease in individuals with hypertension when contrasted with those having prehypertension or normotensive status [135].

## Conclusions

The bidirectional relationship between MBS and periodontitis involves a complex interplay of systemic and oral health factors, mediated by inflammatory responses, oxidative stress, and shared risk factors. Current evidence emphasizes how these conditions mutually influence each other's pathogenesis, with elevated inflammatory markers like TNF-α, IL-1β, and CRP acting as critical links. Characterized by chronic inflammation and microbial dysbiosis, periodontitis contributes to systemic inflammation through the release of pro-inflammatory cytokines and oxidative stress markers. These exacerbate conditions that are hallmark components of MBS, such as IR, hyperglycemia, central obesity, dyslipidemia, and hypertension. Conversely, MBS exacerbates periodontitis by impairing immune responses, promoting dysbiosis, and increasing local and systemic inflammation. Additionally, the altered microbiome in obesity and diabetes further predisposes individuals to periodontitis.

Clinical studies have underscored the need for integrated therapeutic approaches that address both oral and systemic health to mitigate the bidirectional impacts of these diseases. Such therapeutic strategies should focus on controlling inflammation, improving glycemic control, and modifying lifestyle factors. Furthermore, future research should aim to elucidate the specific molecular pathways and biomarkers that mediate this relationship. Longitudinal studies and randomized controlled trials are essential to establish causality and evaluate treatment effectiveness comprehensively. In conclusion, understanding the interplay between periodontitis and MBS highlights the importance of interdisciplinary collaboration in clinical practice. A holistic approach that addresses both periodontal and systemic health components is crucial for improving patient outcomes and reducing the global burden of chronic inflammatory diseases.
